# Surface Modification of Gold Nanoparticle Impacts Distinct Lipid Metabolism

**DOI:** 10.3390/molecules30081727

**Published:** 2025-04-11

**Authors:** Xinyu Ding, Shanshan Liang, Tingfeng Zhang, Minglu Zhang, Hao Fang, Jiale Tian, Jinke Liu, Yuyuan Peng, Lingna Zheng, Bing Wang, Weiyue Feng

**Affiliations:** 1 CAS Key Laboratory for Biomedical Effects of Nanomaterials and Nanosafety, Institute of High Energy Physics, Chinese Academy of Sciences, Beijing 100049, China; xinyu.ding@ihep.ac.cn (X.D.); liangss@ihep.ac.cn (S.L.); zhangtf@ihep.ac.cn (T.Z.); zhangml@ihep.ac.cn (M.Z.); fanghao@ihep.ac.cn (H.F.); jiale.tian@ihep.ac.cn (J.T.); jinke.liu@ihep.ac.cn (J.L.); yuyuan.peng@ihep.ac.cn (Y.P.); zhengln@ihep.ac.cn (L.Z.); 2 University of Chinese Academy of Sciences, Beijing 100049, China; 3 State Key Laboratory of Medicinal Chemical Biology, Key Laboratory of Molecular Drug Research and KLMDASR of Tianjin, College of Pharmacy, Nankai University, Tianjin 300350, China

**Keywords:** gold nanoparticles, surface functionalization, metabolomics, transcriptomics

## Abstract

Gold nanomaterials have garnered significant attention in biomedicine owing to their tunable size and morphology, facile surface modification capabilities, and distinctive optical properties. The surface functionalization of these nanoparticles can enhance their safety and efficacy in nanomedical applications. In this study, we examined the biological effects of gold nanoparticles (GNPs) with three distinct surface modifications (polyethylene glycol, chitosan, and polyethylenimine) in murine models, elucidating their mechanisms of action on hepatic tissue at both the transcriptomic and metabolomic levels. Our findings revealed that PEG-modified GNPs did not significantly alter any major metabolic pathway. In contrast, CS-GNPs markedly affected the metabolic pathways of retinol, arachidonic acid, linoleic acid, and glycerophospholipids (FDR < 0.05). Similarly, PEI-GNPs significantly influenced the metabolic pathways of retinol, arachidonic acid, linoleic acid, and sphingolipids (FDR < 0.05). Through a comprehensive analysis of the regulatory information within these pathways, we identified phosphatidylcholine compounds as potential biomarkers that may underlie the differential biological effects of the three functionalized GNPs. These findings provide valuable experimental data for evaluating the biological safety of functionalized GNPs.

## 1. Introduction

The liver is the major organ responsible for metabolizing carbohydrates, proteins and lipids, as well as for drug biotransformation and detoxification of endogenous and exogenous substances [1]. Additionally, it serves as a major site for the accumulation of nanoparticles (NPs), regardless of exposed route [2,3]. Studies indicate that 30~99% of the administered nanoparticles accumulate in the liver via diverse cell-dependent and cell-independent blood removal pathways [4], making it the key biofiltering organ [5]. NPs exposure may trigger liver injuries, including oxidative stress, DNA damage, inflammation, and metabolism disruption [2], etc. Since the liver is a primary target organ of NPs [4], a comprehensive understanding of NPs effects on the liver metabolism is crucial for biomedical applications.

Gold nanoparticles (GNPs) are considered as promising candidates for healthcare applications due to their distinctive optical and thermal characteristics along with tunable size, shape, and surface chemistry [6]. The GNPs surface can also be easily modified with a diverse array of small molecules such as nucleic acids, peptides, small interfering RNA, antibodies, polymers such as polyethylene glycol (PEG), etc., [7] rendering them highly attractive material for nanomedicine [8,9]. Furthermore, the surface chemistry of GNPs can drastically govern their sub-organ biodistribution, transfer, and clearance profiles within the liver. Studies have demonstrated the pivotal role of surface chemistry in nanomaterials in regulating intracellular lipid metabolism [10]. This modification also impacts their uptake in the liver, particularly in hepatic Kupffer cells and liver sinusoidal endothelial cells (LSECs), hepatocyte, etc. [4]. Our previous study showed that gold nanoparticles (GNPs) capped with the different ligands, such as polyethylene glycol (PEG), chitosan (CS) and polyethylenimine (PEI) capped exhibit distinct profiles of biodistribution, transfer, and clearance in the mouse liver [4,11,12]. Moreover, the deposited GNPs in the liver disrupts the expression of drug-metabolizing enzymes, including drug uptake and efflux transporters, cytochrome P450 (CYP450) isoforms, and phase II metabolized enzymes [13]. To further investigate the effects of NP retention in the liver, the changes at the gene, metabolite, and pathway levels have been identified [4].

In recent years, multi-omics approaches have been increasingly utilized in nanotoxicological research, offering potent tools for investigating nano–bio interactions [14,15,16,17]. Metabolomics analysis may provide insights into hepatocyte dysfunction or cellular stress following the administration of GNPs [17,18,19], as metabolic changes accurately reflect characteristic alterations in biological fluids, cells, and tissues [20,21]. Moreover, metabolomics serves as the upstream input from the environment and the downstream output of the genome [22,23,24,25]. Transcriptomics, which examines the gene expression at the RNA level, involves the study of the total RNA transcribed by a tissue or cell in a specific physiological environment [26]. The integration of omics information can elucidate changes in transcriptome and metabolite expressions induced by NPs. Consequently, the combination of multi-omics technology with bioinformatics presents a cost-effective and reliable approach for the safety assessment of NPs [27,28].

In this study, metabolomics and transcriptomics methodologies were integrated to elucidate the biological effects and trends associated with the accumulation of various surface-functionalized GNPs and their metabolic impact on hepatic tissue. This comprehensive approach revealed the metabolic pathways and corresponding transcriptomes that may be influenced by three distinct types of functional GNPs. The findings contribute significant insights to the biosafety research of GNPs.

## 2. Results and Discussion

### 2.1. Hepatic Metabolites Changes After i.v. Injection of Functional GNPs

The liver, recognized as the largest solid organ in the body, serves as the primary site for the bio-transformation of most exogenous substances and is a significant locus for the accumulation of nanoparticles (NPs), irrespective of the route of exposure [2]. Our previous studies demonstrated that the surface coating, including polyethylene glycol (PEG), chitosan (CS), and polyethylenimine (PEI), significantly influence their sub-organ biodistribution, transfer, and clearance profiles of gold NPs in the liver after intravenous injection in mice [4]. Additionally, we have examined the impact of gold NPs with varying surface coatings on drug-metabolizing enzymes gene expression [5]. It is important to note that protein levels often poorly correlate with mRNA levels due to post-transcriptional regulation mechanism, such as translational efficiency and degradation rates, highlighting the importance of integrating multi-omics data. Here, we investigated the potential alterations of metabolic levels in mice after intravenous injection of GNPs that were surface-modified with functional ligands (PEG-, CS-, PEI-) based on metabolomics and transcriptomics methodologies. As characterized in our previous studies^4^, the synthesized AuNPs exhibit a spherical morphology with a uniform size distribution (~6 nm) (Figure S1). The zeta potentials of PEG-GNPs, CS-GNPS, and PEI-GNPS are −10.2 mV, 11.9 mV, and 5.7 mV, respectively. The successful functionalization of GNPs with PEG, CS, and PEI ligands was confirmed in our previous study through FTIR spectroscopy [4].

**Figure 1:**
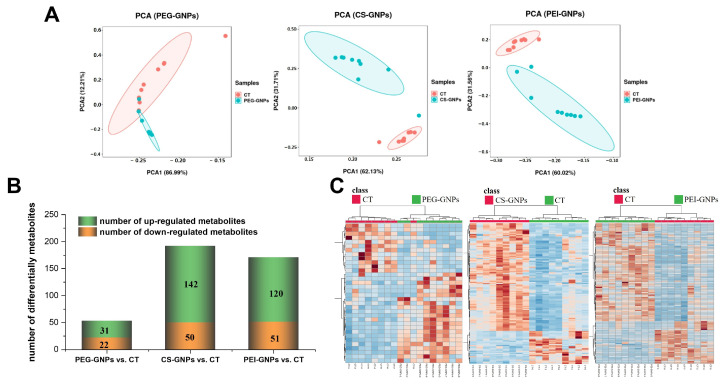
Metabolite statistics and relative expression clustering heatmap of the three functional GNPs with significant content changes between the treated group and the control group, *n* = 9. (**A**) Principal component analysis (PCA) of metabolites between the treated group and the control group; (**B**) the number of differentiated metabolites identified between the treated group and the control group. 53 DAMs in the PEG-GNPs treated group. 192 DAMs in the CS-GNPs treated group. 171 DAMs in the PEI-GNPs treated group; and (**C**) clustered heatmap of relative expression of differential metabolites.

UPLC-ESI-TOF-MS was employed to analyze both aqueous and organic extracts from liver tissues of groups treated with PEG-GNPs, CS-GNPs and PEI-GNPs, as well as a control group, in both positive and negative (ESI+ and ESI−) ion mode. The obtained base peak intensity (BPI) chromatogram (Figure S2) indicated the detection of a variety of small molecule metabolites within the extracts. The BPI demonstrated stable baseline, good resolution and high signal peak intensity for both experimental and vehicle groups within the test period, suggesting that effective separation of endogenous small molecule metabolites in liver tissue. Preliminary identification using Progenesis Q1 revealed the presence of 1771 metabolites in the aqueous phase extract (Figure S2A) under ESI+ mode and 1106 metabolites (Figure S2B) under ESI− mode, respectively. In the organic phase extract, 772 metabolites (Figure S2C) under ESI+ mode and 258 metabolites under ESI- mode (Figure S2D) was detected. Subsequently, a principal component analysis (PCA) model was developed to identify outliers and analyze the natural clustering patterns among the samples. The results (Figure 1A) demonstrated excellent intra-group reproducibility and clear inter-group discrimination in the injected group.

Additionally, orthogonal partial least squares discriminant analysis (OPLS-DA) was employed to compare the differences between the samples from the treated group and the control group. The results indicated (Figure S3) that the three treated groups were clearly separated from the control groups in both positive and negative ion modes, facilitating the screening of differential metabolites. Based on the aforementioned analysis, metabolites meeting the criteria of VIP ≥ 1, FC ≥ 2, *p* < 0.05 and CV < 30% were screened as differentially accumulated metabolites (DAMs). Compared with the control, the PEG-GNPs treated group exhibited 53 DAMs (upregulation of 31 metabolites and downregulation of 22) which were screened, while the CS-GNPs and PEI-GNPs group showed 192 DAMs (upregulation of 142 and downregulation of 50) and 171 DAMs (upregulation of 120 and downregulation of 51), respectively (Figure 1B).

Concurrently, cluster heatmap analysis was conducted on the DAMs identified from the three treated groups. The intensity of most of the markers in the three treated groups was higher than that in the control group (Figure 1C). Comparatively, it was found that the identical DAMs among the three groups accounted for 5.51% of the total DAMs. Notably, the proportion of common DAMs between the CS-GNPs and PEI-GNPs treated groups was as high as 42.64%. However, the percentage of common DAMs between the PEG-GNPs group and either of the other two groups did not exceed 7.5%.

### 2.2. Signaling Pathway Analysis of Differentially Accumulated Metabolites

Phospholipids constitute the primary components of cell membranes, encompassing glycerophospholipids and sphingomyelin. Glycerophospholipids include phosphatidylcholine (PC), phosphatidylethanolamine (PE), phosphatidylserine (PS) and phosphatidylinositol (PI), with PE and PC comprising over 75% of the phospholipid content [29]. PC, also referred to as lecithin, is rich in unsaturated fatty acids capable of combining with free radicals in the body to neutralize them and it exhibits certain anti-inflammatory properties [30,31]. PE, also known as cephalin, ranks as the second most abundant phospholipid in the liver following PC [32]. PE is characterized by a high content of unsaturated fatty acids in its structure [29] and can undergo interconversion with PC in the liver [32,33]. In this study, it is noteworthy that the majority of these DAMs in the treated groups were lipid compounds. PEG-, CS- and PEI-GNPs disrupted the glycerophospholipid metabolic pathway, with CS- and PEI-GNPs specifically involved in phosphocholine (C04230) [34].

**Figure 2:**
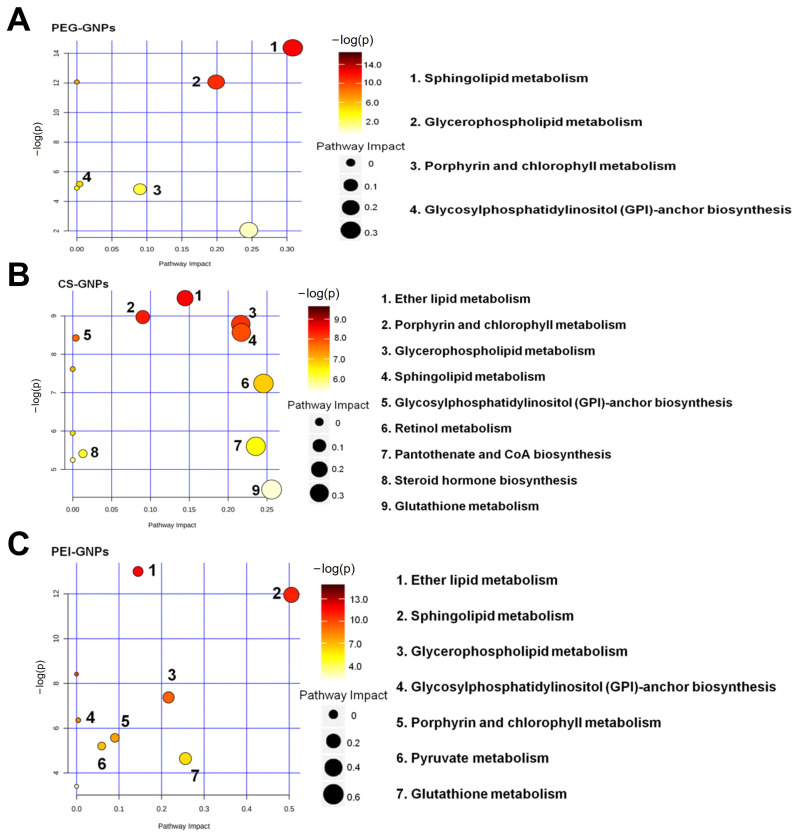
Bubble plot of KEGG pathway enrichment for differential metabolites in the treated groups (compared to control, *n* = 9). (**A**) PEG-GNPs treated group. (**B**) CS-GNPs treated group. (**C**) PEI-GNPs treated group.

**Figure 3:**
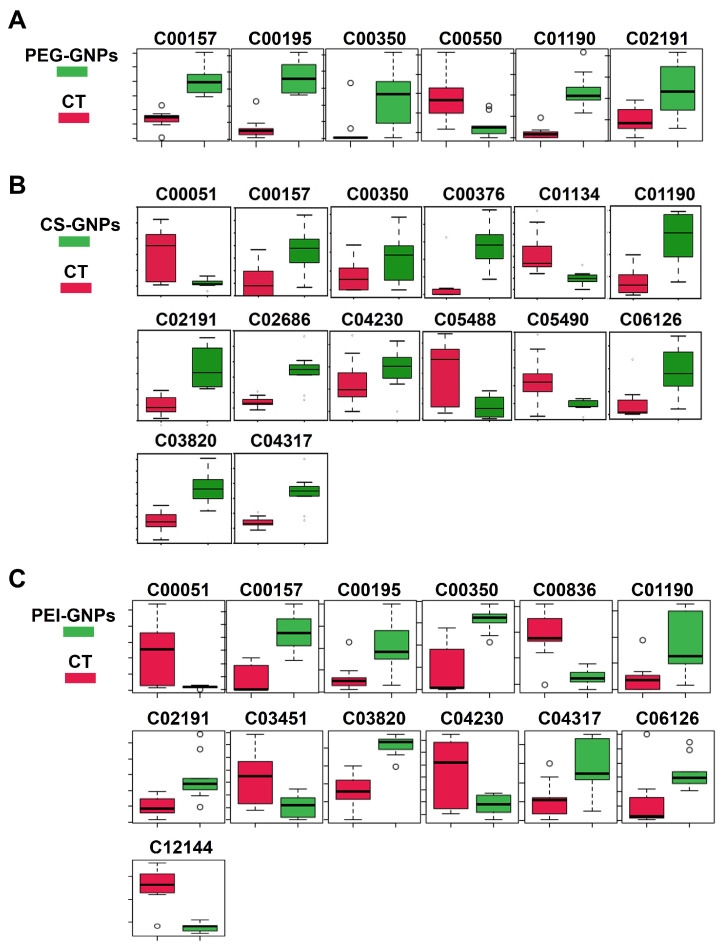
Comparison of liver tissue metabolites in the treated and the control groups, *n* = 9. (**A**) PEG-GNPs treated group vs. the control. (**B**) CS-GNPs treated group vs. the control. (**C**) PEI-GNPs treated group vs. the control. The circles outside the box in a box plot (box-and-whisker plot) typically represent outliers, not the interquartile range (IQR).

Using databases such as HMDB, KEGG and MetaboAnalyst, we identified metabolic pathways of DAMs associated with alterations in mouse liver tissue after injection of PEG-GNPs, CS-GNPs and PEI-GNPs. Under the conditions of *p* < 0.05 and q < 0.05, significantly altered signaling pathways were independently screened from the three treated groups (Figure 2). The results showed that compared with the control, DAMs in PEG-GNPs group were mainly enriched in sphingolipid metabolism, glycerophospholipid metabolism, glycosylphosphatidylinositol anchor biosynthesis, and porphyrin and chlorophyll metabolism (Figure 2A). For CS-GNPs, the enrichment was predominantly involved in ether lipid metabolism, sphingolipid metabolism, glycerophospholipid metabolism, glycosylphosphatidylinositol biosynthesis, steroid hormone biosynthesis, porphyrin and chlorophyll metabolism, retinol metabolism, pantothenate and CoA biosynthesis and glutathione metabolism (Figure 2B). DAMs in PEI-GNPs were enriched in ether lipid metabolism, sphingolipid metabolism, glycerophospholipid anchor metabolism, glycosylphosphatidylinositol biosynthesis, porphyrin and chlorophyll metabolism, pyruvate metabolism and glutathione metabolism (Figure 2C). Table S1 presents the metabolic pathways and associated metabolites influenced by the three treatment groups in liver tissue. In conjunction with the comparative graphs, it is evident that the metabolites are more significantly correlated with the effects on metabolic pathways in the CS-GNPs and PEI-GNPs groups. CS-GNPs and PEI-GNPs induced more obvious metabolic changes than those in the PEG-GNPs treated group. Among them, changes in sphingolipid metabolism and glycerophospholipid metabolism are more significant, including glucosylceramide (C01190), phosphatidylcholine (PC, C00157), phosphatidylethanolamine (PE, C00350) and protoporphyrin (C02191) (Figure 3). Compared to the control, C01190 levels were significantly elevated in all three exposed groups. Notably, CS- and PEI-GNPs induced more pronounced metabolic alterations than PEG-GNPs, affecting key metabolites such as glutathione (C00051), 1-alkyl-2-acetyl-sn-glycerol (C03820), and digalactosylceramide (C06126).

### 2.3. Transcriptomic and Metabolomics Integration Analysis

**Figure 4:**
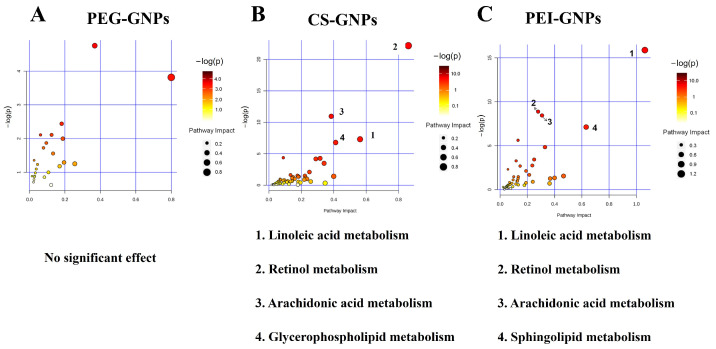
Bubble map of KEGG pathway enrichment involved in the integration of differentially expressed genes and metabolites in mouse liver tissue after tail vein injection of three functional GNPs, respectively. (**A**) PEG-GNPs treated group. (**B**) CS-GNPs treated group. (**C**) PEI-GNPs treated group, *n* = 9.

In our previous study, we investigated the effects of the deposited GNPs in the liver on the expression of drug-metabolizing enzymes and lipid homeostasis [5]. Based on these findings, we performed an integrated analysis of transcriptomics and metabolomics to further elucidate the molecular mechanisms involved. Differentially expressed genes (DEGs; selection criteria: FC ≥ 1.5, *p* < 0.05, average strength > 7) and differentially abundant metabolites (DAMs; VIP ≥ 1, FC ≥ 2, *p* < 0.05, QC sample CV < 30%) were analyzed using MetaboAnalyst, HMDB, and KEGG databases. We then constructed metabolic pathways associated with DEGs and DAMs in mouse liver tissue following the injection of PEG-GNPs, CS-GNPs and PEI-GNPs and screened significantly altered signaling pathways (*p* < 0.05, q < 0.05, weight coefficient > 0). The results indicate that no signaling pathways met the screening criteria in the PEG-GNPs group (Figure 4). In contrast, the CS-GNPs disrupted several key signaling pathways, including retinol metabolism, arachidonic acid metabolism, linoleic acid metabolism, and glycerophospholipid metabolism, while, PEI-GNPs modulated signaling pathways such as retinol metabolism, arachidonic acid metabolism, linoleic acid metabolism, and sphingolipid metabolism. As shown in Table 1, the MetaboAnalyst analysis summarizes genes associated with the enrichment of metabolic signaling pathways.

**Figure 5:**
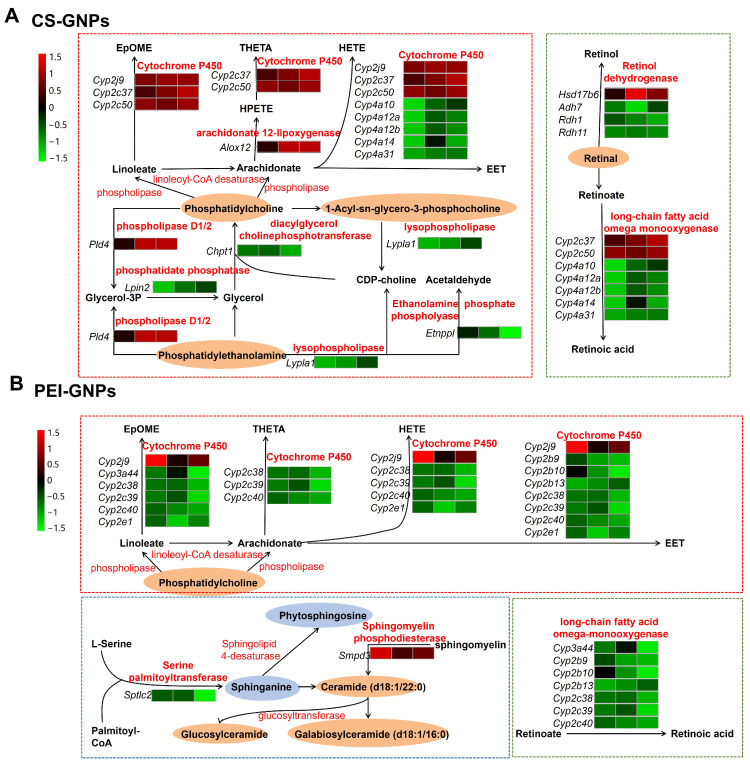
Transcriptomic and metabolomic analysis of metabolic signaling pathways in liver tissue KEGG map. (**A**) CS-GNPs treated group. (**B**) PEI-GNPs treated group.

Further analysis was performed on the metabolite changes associated with CS-GNPs and PEI-GNPs (Figure 5). CS-GNPs induced significant alterations in glycerophospholipid, linoleic acid, and retinol metabolism. Within the glycerophospholipid pathway, there was a notable increase in the expression of phosphatidylcholine (PC), phosphatidylethanolamine (PE), and phosphocholine. Additionally, CS-GNPs influenced the gene expression of *Chpt*1, *Lpin*2, *Pld*4, *Lypla*1, and *Etnppl*, which are related to glycerophospholipid metabolism. Both the CS-GNPs and PEI-GNPs treated groups significantly affected linoleic acid metabolism. CS-GNPs activated linoleic acid metabolism through *Cyp*2*c* genes (*Cyp*2*c*50, *Cyp*2*c*37), *Cyp*2*j* (*Cyp*2*j*9), *Cyp4a* (*Cyp*4*a*10, *Cyp*4*a*12*b*, *Cyp*4*a*14, *Cyp*4*a*12*a*) subfamilies and *Alox12*. In contrast, PEI-GNPs impacted linoleic acid metabolism through genes in the *Cyp*2*c, Cyp*2*j, Cyp*2*e* and *Cyp*3*a* subfamilies, such as *Cyp*2*b*10, *Cyp*2*b*13, *Cyp*2*b*9, *Cyp*2*c*38, *Cyp*2*c*39, *Cyp*2*c*40, *Cyp*2*j*9, *Cyp*2*e*1. Linoleic acid is involved in numerous immune responses and cellular growth processes in the body, particularly through its ability to bind with cholesterol to form esters, facilitating the degradation of cholesterol into bile acids for excretion [35,36]. The metabolic products of the activated pathways in both groups were the linoleic acid epoxides (EpOMEs). EpOMEs metabolites generally exhibit pro-inflammatory effects, with in vitro experiments demonstrating their ability to promote the expression of pro-inflammatory cytokines in macrophages [37]. Regarding arachidonic acid metabolism, CS-GNPs modulated the process of arachidonic acid generation by PC through several genes (enzymes) in the *Cyp4a* and *Cyp2j* subfamilies, while the PEI-GNPs primarily affected this process through genes (enzymes) in the *Cyp2b* and *Cyp2c* subfamilies. Both CS-GNPs and PEI-GNPs also activated the LOXs lipoxygenase pathway to produce hydroperoxyeicosatetraenoic acid (HPETE), hydroxyeicosatetraenoic acid (HETEs), as well as the CYP monooxygenase pathway to produce HETEs and epoxidized eicosatrienoic acid (EETs).

Following the integrated transcriptome and metabolome analysis, both the CS-GNPs and PEI-GNPs also significantly affected retinol metabolism. Retinol, a form of vitamin A, exhibits antioxidant properties and is also associated with immune and inflammatory responses [38,39]. The differential metabolite retinaldehyde was identified in the CS-GNPs group. Retinaldehyde is classified as a non-enzymatic antioxidants and its conversion to retinol is reversible [40]. The DEGs involved in retinol metabolism include *Adh*7, *Hsd*17*b*6, *Rdh*11, *Rdh*1, *Cyp*4*a*10, *Cyp*4*a*12*b*, *Cyp*4*a*14, *Cyp*4*a*12*a*, *Cyp*4*a*31, *Cyp*2*c*50, *Cyp*2*c*37. The genes *Adh*7, *Hsd*17*b*6, *Rdh*11, and *Rdh*1 were mainly responsible for transforming oxidized retinol to retinaldehyde. Alterations in the monooxygenase’s genes of the *Cyp*4*a* and *Cyp*2*c* subfamilies influenced the metabolic process of all-trans-retinoate to all-trans-18-hydroxy-retinoic acid. Thus, the retinol metabolism activated by CS-GNPs is involved in the oxidation of retinol into retinaldehyde, leading to an increase in retinaldehyde content and the activation of signaling pathways in the subsequent retinol ester hydrolysis process. In contrast, the PEI-GNPs affected three metabolic processes of all-trans-retinoate through several genes (enzymes) of the *Cyp*3*a*, *Cyp*2*b* and *Cyp*2*c* subfamilies, including the process of hydrolysis of all-trans-retinyl ester to all-trans-4-hydroxyretinoic acid, followed by its conversion to all-trans-4-oxoretinoic acid; the process of hydrolysis of all-trans-retinyl ester to all-trans-18-hydroxyretinoic acid; and the transport and metabolism study of all-trans-retinyl ester metabolizing to all-trans-5, 6-epoxy-5, and 6-dihydroretinoic acid. The gene expression of *Cyp*2*b*10, *Cyp*2*b*13 and *Cyp*2*b*9 significantly decreased, particularly those of *Cyp*2*b*13 and *Cyp*2*b*9. Thus, the PEI-GNPs activated the signaling pathways of retinol ester hydrolysis process.

Notably, the PEI-GNPs significantly affected the sphingolipid metabolism signaling pathway (Figure 5). Sphingolipids, the second-most abundant class of lipids after phospholipids, possess a backbone of long-chain sphingosine. As essential components of membrane structures, sphingolipids play crucial roles in cell growth, differentiation, and apoptosis processes, participating in numerous signaling pathways [41]. The metabolites affected in sphingolipid metabolism include ceramide, galactosylceramide, glucosylceramide, and sphingosine, with the involved differentially expressed genes (DEGs) being *Sptlc*2 and *Smpd*3. Ceramide, central to sphingolipid metabolism, serves as a second messenger of lipids and is also involved in many processes of cell growth, differentiation, senescence and apoptosis [42]. Glycosphingolipids can be degraded into ceramide by glycosyl hydrolases, and ceramide is then converted into glucosylceramide under the action of glucosyltransferase, a process that also leads to an increase in the level of glucosylceramide. Thus, the PEI-GNPs interfered with sphingolipid metabolism signaling pathways, including the ceramide biosynthesis pathway (M00094) and the lactosylceramide biosynthesis pathway (M00066).

The integrated analysis of DEGs and differential metabolites indicates that the PEG-GNPs do not interfere with any signaling pathways meeting the specified criteria. In contrast, CS-GNPs disrupt pathways related to retinol metabolism, arachidonic acid metabolism and linoleic acid metabolism. Similarly, PEI-GNPs interfered with retinol metabolism, arachidonic acid metabolism, linoleic acid metabolism, and sphingolipid metabolism. It is apparent that these affected pathways are all linked to lipid metabolism. Particularly, CS-GNPs and PEI-GNPs exhibit overall similarities in the pathways they impact. Through network interaction analysis, key differential genes and metabolites have been identified. Our findings suggest that phosphatidylcholine compounds constitute a class of potential key metabolites responsible for inducing different metabolic mechanisms. The combined transcriptomic and metabolomic analysis provide a foundation for in-depth research into the metabolic regulatory mechanisms associated with different functionalized GNPs and provides comprehensive and systematic information for the study of GNPs’ biosafety.

## 3. Materials and Methods

### 3.1. Reagents and Materials

The synthesis of functional gold nanoparticles (GNPs), including PEG-GNPs, CS-GNPs, and PEI-GNPs, was described in our previous work, with detailed procedures provided in the Supporting Information. Methanol (HPLC grade, ≥99.9%) was purchased from Dikma Limited (Beijing, China), and dichloromethane (DCM, GC grade, ≥99.9%) was obtained from OceanPAK (Gothenburg, Sweden). Ultrapure deionized water (18.2 MΩ) from a Milli-Q system (Merck KGaA, Darmstadt, Germany) was used in all experiments.

### 3.2. Animal Experiment

Male CD-1 (ICR) mice (7 weeks old, 20 ± 1 g body weight) were obtained from Beijing Vital River Laboratory Animal Technology Co., Ltd. (Beijing, China). All animal experiments were approved by the Committee on the Ethics of Animal Experiments of the Institute of High Energy Physics, Chinese Academy of Sciences, and conducted in accordance with the guidelines of the Chinese Society of Toxicology and Ethics Committee. The mice were housed in a conventional animal facility maintained at 25 ± 2 °C and 60 ± 2% humidity, with a 12 h light/dark cycle. They were provided with a standard commercial pellet diet and deionized water ad libitum. Animals were acclimatized for at least three days prior to the experiments.

For metabolomic and transcriptomic analysis, the mice were randomly divided into four groups, with 9 mice in each group, 200 μL of freshly prepared suspensions of PEG-GNPs, CS-GNPs, and PEI-GNPs were intravenously injected into the mice via the tail vein at doses of 5.0, 5.0, and 0.8 μg/g body weight, respectively. The control group mice were injected with 0.9% NaCl solution via the tail vein. Mice injected with saline solution served as the vehicle control group. The mice were sacrificed 7 days after a single injection of GNPs. Liver tissue was collected, quickly rinsed with RNase-free water to remove blood, blotted dry, and then stored at −80 °C.

### 3.3. Hepatic Metabolomic Analysis

Liver metabolite extraction: liver samples (50 mg) were homogenized utilizing a handheld homogenizer (Tissue-Tearor Superfine, Biodex, Shirley, NY, USA) in 1.5 mL of methanol solution for aqueous extraction, followed by organic extraction with 1.5 mL of dichloromethane/methanol (3:1). Lansoprazole, clozapine, and carbamazepine, each at a concentration of 200 μg/mL in the extraction solution, were employed as internal standards. The homogenates were centrifuged at 16,000× *g* for 10 min, after which the supernatants were collected in Eppendorf tubes and desiccated in a SpeedVac vacuum concentrator (Thermo Fisher Scientific, Waltham, MA, USA). The desiccated extracts were resuspended in methanol/water (1:1) prior to UPLC-MS analysis.

UPLC-ESI-QTOFMS analysis: the aqueous and organic extracts for metabolomic analysis were subjected to ultra-performance liquid chromatography equipped with quadrupole time-of-flight mass spectroscopy (UPLC-ESI-QTOFMS, Waters, Santa Clara, CA, USA). Mass spectrometry was conducted in positive (ESI+) and negative (ESI−) ionization modes separately. The optimized electrospray ionization (ESI) conditions were as follows: source and desolvation temperature at 100 °C and 400 °C, respectively, cone gas flow 50 L/h, desolvation gas flow 800 L/h, capillary voltage 3000 V for ESI+ and 2500 V for ESI− modes, and cone voltage 40 V. The instrument was configured to acquire in a *m*/*z* range of 50–1200 with a scan time of 0.5 s and an interscan delay of 0.1 s. An Acquity HSS T3 column (2.1 mm × 100 mm, 1.7 μm, Waters Corp, Milford, MA, USA) was utilized with a 0.4 mL/min flow rate under an aqueous 0.1% (*v*/*v*) formic acid/methanol gradient for the aqueous extract chromatographic separation. For the organic extract separation, an Acquity BEH C8 (2.1 × 100 mm, 1.7 μm, Waters Crop) was employed with a 0.4 mL/min flow rate under a 75% methanol/isopropanol (*v*/*v* = 7:3) gradient. The quality analysis was described in Supporting Information.

Data processing: The mass spectral data were pre-processed utilizing Progenesis QI software v2.0 (Waters Corp., Milford, MA, USA) for peak extraction, alignment, and normalization. MetaScope plug was utilized for the in-house library with accurate mass, fragmentation pattern, and retention time for database search. Subsequently, the data were transferred into EZinfo 2.0 software (Waters Corp., Milford, MA, USA) to perform PCA and OPLS-DA analysis. The variables with variable importance in projection (VIP) value above 1.0 and *p* value below 0.05 were retained in subsequent metabolomic analysis. Differentially accumulation metabolites (DAMs) were selected as those with a |fold change| > 2, and *p* < 0.01 (treated vs. control mice). The online Human Metabolome Database (http://www.hmdb.ca/, accessed on 9 October 2019) was also utilized for metabolite searching and identification. MetaboAnalyst 4.0 was employed for pathway analysis according to the pathways defined in KEGG (www.genome.jp/kegg/, accessed on 8 November 2019).

### 3.4. Hepatic Transcriptomic Analysis

Transcriptomic analysis method has been described in our previous study [4]. In brief, 60 ng of total RNA was amplified and labeled with Cy3 using the Agilent Low Input Quick Amp Labeling One Color Kits (Santa Clara, CA, USA) and then hybridized to the Agilent SurePrint G3 Mouse Gene Expression v2 8 × 60 K microarray (Santa Clara, CA, USA) according to the manufacturer’s specifications. Raw data were normalized using a quantile algorithm in the R/Bioconductor limma package. To detect expression differences between the treated and vehicle groups, a moderated *t*-test was applied. Differentially expressed genes (DEGs) were selected based on a fold change > 1.5 or <−1.5, with a significance level of *p* < 0.01 (treated vs. control mice). Enrichment analysis of DEGs were conducted using the Gene Ontology Consortium (GO: https://www.geneontology.org/, accessed on 8 September 2019) and the KEGG public pathway resource.

### 3.5. Integrated Analysis of Transcriptomic and Metabolomic: Pathway-Based Integration

The primary method for integrating transcriptomic and metabolomic data is through pathway-based integration. Based on the pathway analysis results of differentially accumulated metabolites (DAMs) and differentially expressed genes (DEGs), genes and metabolites involved in the same pathway were screened, followed by comprehensive analysis of the selected data. During this process, ID conversion methods were employed. In this experiment, gene and metabolite IDs were converted according to the KEGG format and mapped to the KEGG database, ultimately generating integrated pathway maps of genes and metabolites.

The integration process first involved screening DEGs that met the criteria of FC ≥ 1.5, *p* < 0.05, and an average intensity value > 7. Similarly, DAMs were screened based on the criteria of VIP ≥ 1, FC ≥ 2, *p* < 0.05, and a QC sample CV < 30%. Subsequently, MetaboAnalyst 4.0 was used to integrate the DEGs and DAMs, constructing interaction pathway maps that included metabolites, DEGs, and their associated regulatory enzymes. Finally, representative DEGs and DAMs were identified for further analysis and discussion. In this study, pathways meeting the criteria of *p* < 0.05, q < 0.05, and a weight coefficient > 0 were selected as representative metabolic pathways for analysis.

### 3.6. Statistical Analysis

A Student’s *t*-test was used to compare biochemical data, metabolites or gene expressions between GNPs treated and vehicle control groups. The supervized OPLS-DA was carried out to discover the best discriminators between the groups. All other statistical tests (hierarchical clustering and two-way ANOVA) were performed using MeV 4.9 open-source software. The differences were considered statistically significant at *p* < 0.05.

## 4. Conclusions

This study has demonstrated that surface-functionalized gold nanoparticles (GNPs) induce significant metabolomic changes in mouse liver tissue. A combined metabolomic and transcriptomic analysis elucidated the signaling pathways and genes affected by GNPs, resulting in an interaction network diagram of metabolites and gene expression in mouse liver tissue following GNPs injection. Notably, PEG-GNPs did not result in any pathways with significant differences; however, the CS-GNPs-treated group exhibited significant effects on the retinol metabolism, arachidonic acid metabolism, linoleic acid metabolism, and glycerophospholipid metabolism pathways. The PEI-GNPs demonstrated significant effects on retinol metabolism, arachidonic acid metabolism, linoleic acid metabolism, and sphingolipid metabolism pathways. These findings elucidate the potential mechanisms and effects of surface chemically modified GNPs on various biological processes in mice and establish a foundation for in-depth studies of the metabolic regulatory mechanisms involved in different functionalized GNPs. Furthermore, they provide systematic and comprehensive information for research on the biological safety of GNPs.

## Figures and Tables

**Table 1:** Differently expressed gene and metabolite.

Pathway Name	Hits	Total	*p*	FDR	Impact	Matched Features
PEG-GNPS						
Sphingolipid metabolism	3	58	0.008593	0.722	0.36842	cpd:C00550; cpd:C00195; cpd:C01190
Glycosphingolipid biosynthesis-globo and isoglobo series	2	31	0.021983	0.923	0.8	mmu:239559; mmu:26879
**CS-GNPs**						
Retinol metabolism	12	44	2.34 × 10^−10^	1.97 × 10^−8^	0.86047	cpd:C00376; mmu:11529; mmu:27400; mmu:17252; mmu:107141; mmu:13096; mmu:107605; mmu:13117; mmu:13118; mmu:13119; mmu:277753; mm:666168
Arachidonic acid metabolism	10	79	1.76 × 10^−5^	7.40 × 10^−4^	0.38462	cpd:C00157; mmu:107141; mmu:13096; mmu:74519; mmu:13117; mmu:13118; mmu:13119; mmu:277753; mmu:666168; mmu:11684
Linoleic acid metabolism	4	17	0.000675	1.89 × 10^−2^	0.5625	cpd:C00157; mmu:107141; mmu:13096; mmu:74519
Glycerophospholipid metabolism	8	86	0.001137	2.39 × 10^−2^	0.41176	cpd:C00350; cpd:C00157; cpd:C04230; mmu:212862; mmu:64898; mmu:104759; mmu:18777; mmu:71760
**PEI-GNPS**						
Linoleic acid metabolism	7	17	1.26 × 10^−7^	1.06 × 10^−5^	1.0625	cpd:C00157; mmu:13097; mmu:13098; mmu:13099; mmu:74519; mmu:13106; mmu:337924
Retinol metabolism	7	44	0.000139	5.84 × 10^−3^	0.27907	mmu:337924; mmu:13088; mmu:13089; mmu:13094; mmu:13097; mmu:13098; mmu:13099
Arachidonic acid metabolism	9	79	0.000215	6.02 × 10^−3^	0.30769	cpd:C00157; mmu:13088; mmu:13089; mmu:13094; mmu:13097; mmu:13098; mmu:13099; mmu:74519; mmu:13106
Sphingolipid metabolism	7	58	0.000807	1.69 × 10^−2^	0.63158	cpd:C00836; cpd:C00195; cpd:C06126; cpd:C01190; cpd:C12144; mmu:58994; mmu:20773

## Data Availability

All data generated or analyzed during this study are available from corresponding authors on reasonable request.

## Supplementary Materials

The following supporting information can be downloaded at: https://www.mdpi.com/article/10.3390/molecules30081727/s1, Figure S1: TEM images of functional GNPs. The scale bar represents 10 nm; Figure S2: Representative LC-MS total ion chromatograms (TIC) of vehicle, PEG-GNP, CS-GNP, and PEI-GNP treated liver samples under positive and negative ion modes; Figure S3: Two-dimensional OPLS-DA score plot of metabolic patterns in the PEG-GNPs, CS-GNPs, and PEI-GNPs injection groups; Table S1: Differently expressed gene and metabolite.
