# Supplementary material for: Surface Modification of Gold Nanoparticle Impacts Distinct Lipid Metabolism
Source: Molecules. 2025 Apr 11;30(8):1727. doi: 10.3390/molecules30081727 (PMC12029855; doi:10.3390/molecules30081727)
Supplement: Supplementary file 1 [file molecules-30-01727-s001.zip › molecules-3493556-supplementary.pdf]

Supporting Information (SI)

Surface Modification of Gold Nanoparticle Impacts Distinct Lipid Metabolism

Xinyu Ding[1, #], Shanshan Liang[1,2, #], Tingfeng Zhang[1,2], Minglu Zhang[1,3], Hao Fang[1,2], Jiale Tian[1,2], Jinke Liu[1], Yuyuan Peng[1], Lingna Zheng[1], Bing Wang[1,*], Weiyue Feng[1,*]

1 CAS Key Laboratory for Biomedical Effects of Nanomaterials and Nanosafety, Institute of High Energy Physics, Chinese Academy of Sciences, Beijing 100049, China

2 University of Chinese Academy of Sciences, Beijing 100049, China

3 State Key Laboratory of Medicinal Chemical Biology, College of Pharmacy, Key Laboratory of Molecular Drug Research and KLMDASR of Tianjin, Nankai University, Tianjin, 300350, China

# Xinyu Ding and Shanshan Liang contributed equally to this work.

* Corresponding author: Bing Wang, Email: wangbing@ihep.ac.cn; Weiyue Feng, Email: fengwy@ihep.ac.cn

Materials and Methods

Reagents and materials

Hydrogen tetrachloroaurate trihydrate ($HAuCl_4 \cdot 3H_2O$, 99.99%) was procured from Sinopharmacy Company (Beijing, China); and trisodium citrate dihydrate ($Na_3C_6H_5O_7$, 99%) was obtained from Alfa Aesar (Ward Hill, MA, USA). Methoxy PEG Thiol (mPEG-SH, M.W. 5000 ± 500, ≥ 90%) was acquired from Jenkem Technology Co. Ltd. (Beijing, China); ethylene imine polymer [$(CH_2CH_2NH)_n$, M.W. 10,000, ≥ 99%] from Aladdin Chemical Co. Ltd; and glycol chitosan (≥ 60%) from Sigma Aldrich.

Synthesis of PEG-GNPs, CS-PNGs and PEI-GNPs

A specific quantity of 1% wt. $HAuCl_4$ and 0.1% $AgNO_3$ solution was combined, followed by the addition of 2% citrate aqueous solution. The mixture was introduced into vigorously stirring deionized water under reflux conditions until the solution's color transitioned to wine red. The resultant Cit-GNP solution was allowed to cool to room temperature. For PEGylation of GNPs, the Cit-GNPs were re-suspended in deionized water, and 12.5 mg/mL PEG-5000 was added, immediately vortexed, and subsequently incubated at 4°C for 8 h. For chitosan (CS) modification, 0.75 mg/mL CS solution was introduced into the Cit-GNP suspension solution under vigorous stirring for 30 min at room temperature. For PEI-GNPs synthesis, 0.4 mg/mL PEI solution was added to the Cit-GNP suspension solution under vigorous stirring at room temperature for 30 min. The resulting functional GNPs were centrifuged at 16,000 g multiple times to remove unreacted chemical compounds. The obtained GNPs were re-dispersed in saline solution and stored at 4°C.

Quantitative analysis of UPLC-ESI-QTOF-MS

The instrument was calibrated prior to analysis utilizing a 0.5 mM sodium formate solution. Data were acquired in centroid mode in both ESI+ and ESI- modes. Leucine enkephalin (MW = 555.62 Da) was employed as a lock mass at a concentration of 200 pg/μL in acetonitrile/water (50:50) to ensure accuracy during the MS analysis. A quality control sample (QC) was prepared by combining 20 L aliquots from each of the samples under analysis. The QC samples were initially injected to assess the quality of the analytical run, including the intensity of the total ion chromatograms (TICs), replicates of the QC samples, precision of the peaks, and other relevant parameters. During the global metabolomic analysis, a pool QC sample was run after every five liver extract samples to monitor the stability of the retention time, signal intensity, and mass accuracy of the UPLC-MS system.

The cut-off for retention time match was 0.5 min; and spectral similarity was greater than 30% fragmentation match in Progenesis QI. Peaks that exhibited a coefficient of variation (CV) of less than 30% in pool QC samples were retained for further analysis. Additionally, manual verification of each detected feature was conducted for the selection of appropriate peaks.

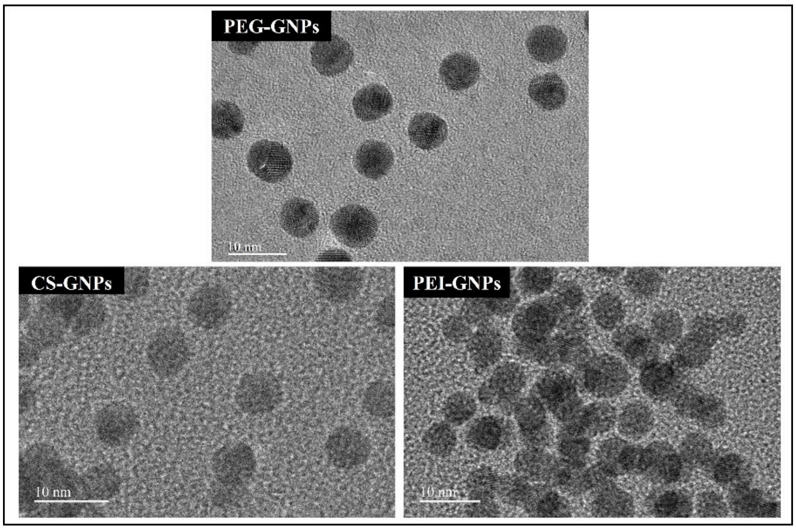

Figure S1. TEM images of functional GNPs. The scale bar represents 10 nm**.**

A

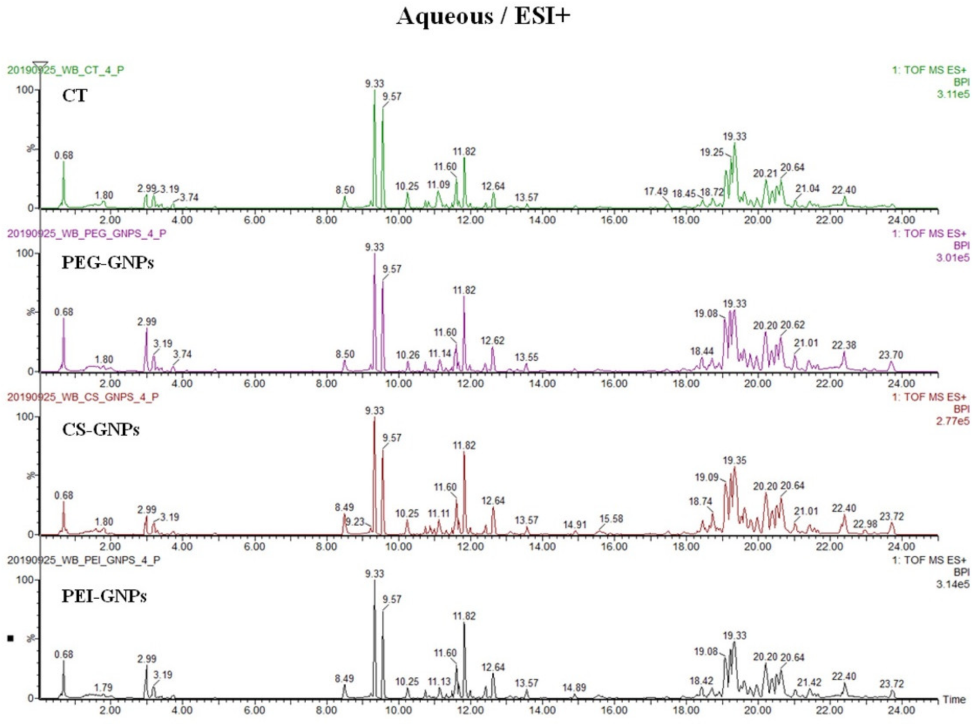
Aqueous / ESI+
20190925_WB_CT_4_P
1: TOF MS ES+
BPI
3.11e5
CT
20190925_WB_PEG_GNPS_4_P
1: TOF MS ES+
BPI
3.01e5
PEG-GNPs
20190925_WB_CS_GNPS_4_P
1: TOF MS ES+
BPI
2.77e5
CS-GNPs
20190925_WB_PEI_GNPS_4_P
1: TOF MS ES+
BPI
3.14e5
PEI-GNPs
Time

B

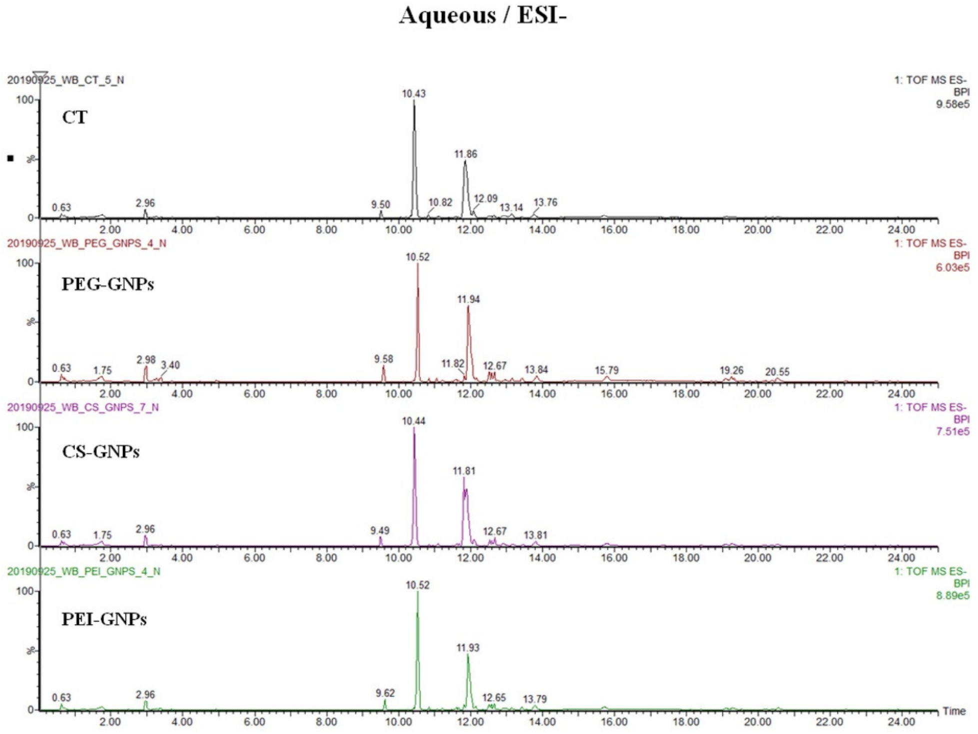
Aqueous / ESI-
20190925_WB_CT_5_N
1: TOF MS ES-
BPI
9.58e5
CT
20190925_WB_PEG_GNPS_4_N
1: TOF MS ES-
BPI
6.03e5
PEG-GNPs
20190925_WB_CS_GNPS_7_N
1: TOF MS ES-
BPI
7.51e5
CS-GNPs
20190925_WB_PEI_GNPS_4_N
1: TOF MS ES-
BPI
8.89e5
PEI-GNPs
Time

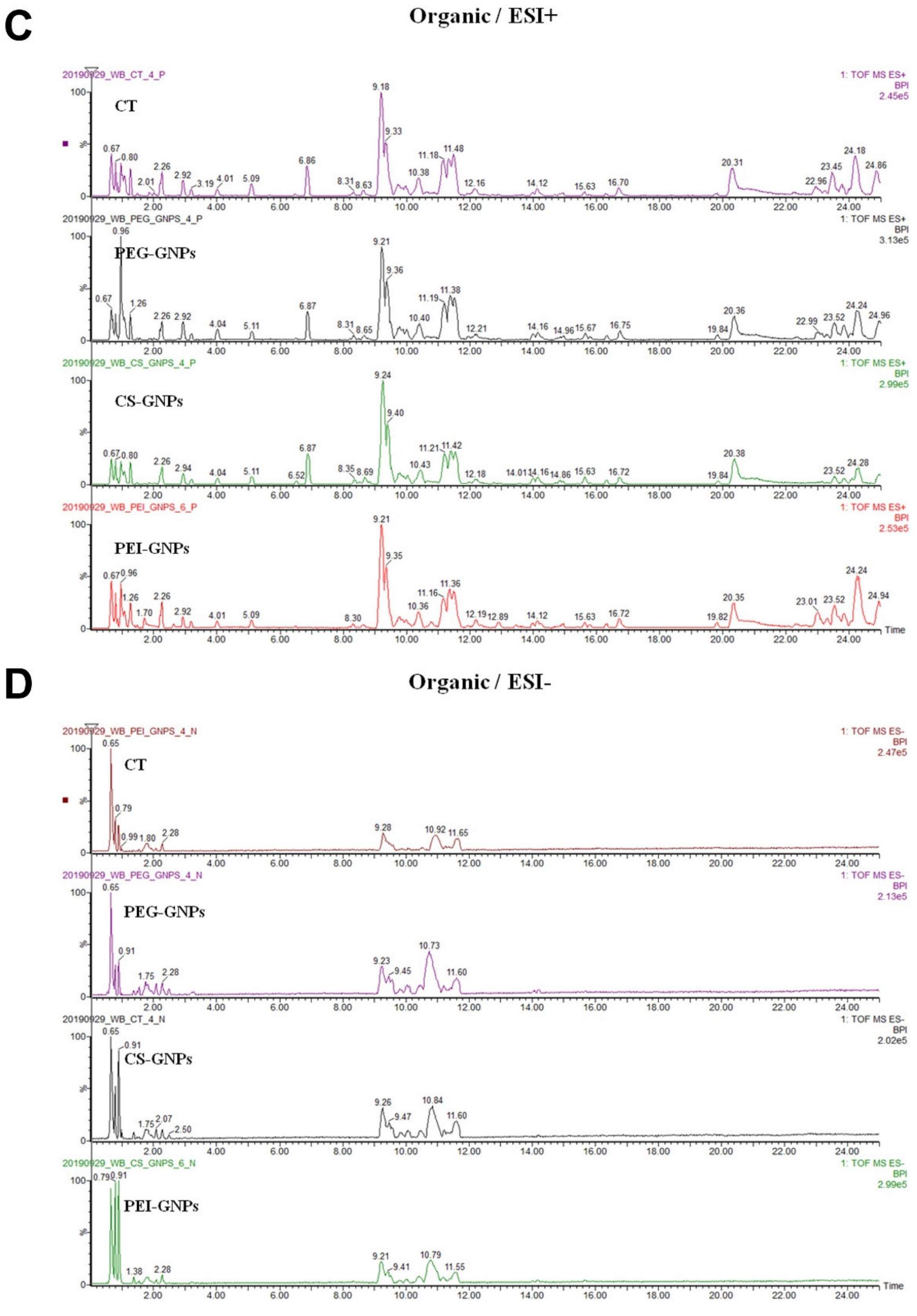

Figure S2. Representative LC-MS total ion chromatograms (TIC) of vehicle, PEG-GNP, CS-GNP, and PEI-GNP treated liver samples under positive and negative ion modes. (A, B) The aqueous extract chromatograms utilizing an Acquity HSS T3 column (2.1 × 100 mm, 1.7 μm, Waters Crop.) for separation. (C, D) The organic extract chromatograms employing an Acquity BEH C8 (2.1 × 100 mm, 1.7 μm, Waters Crop.) for separation.

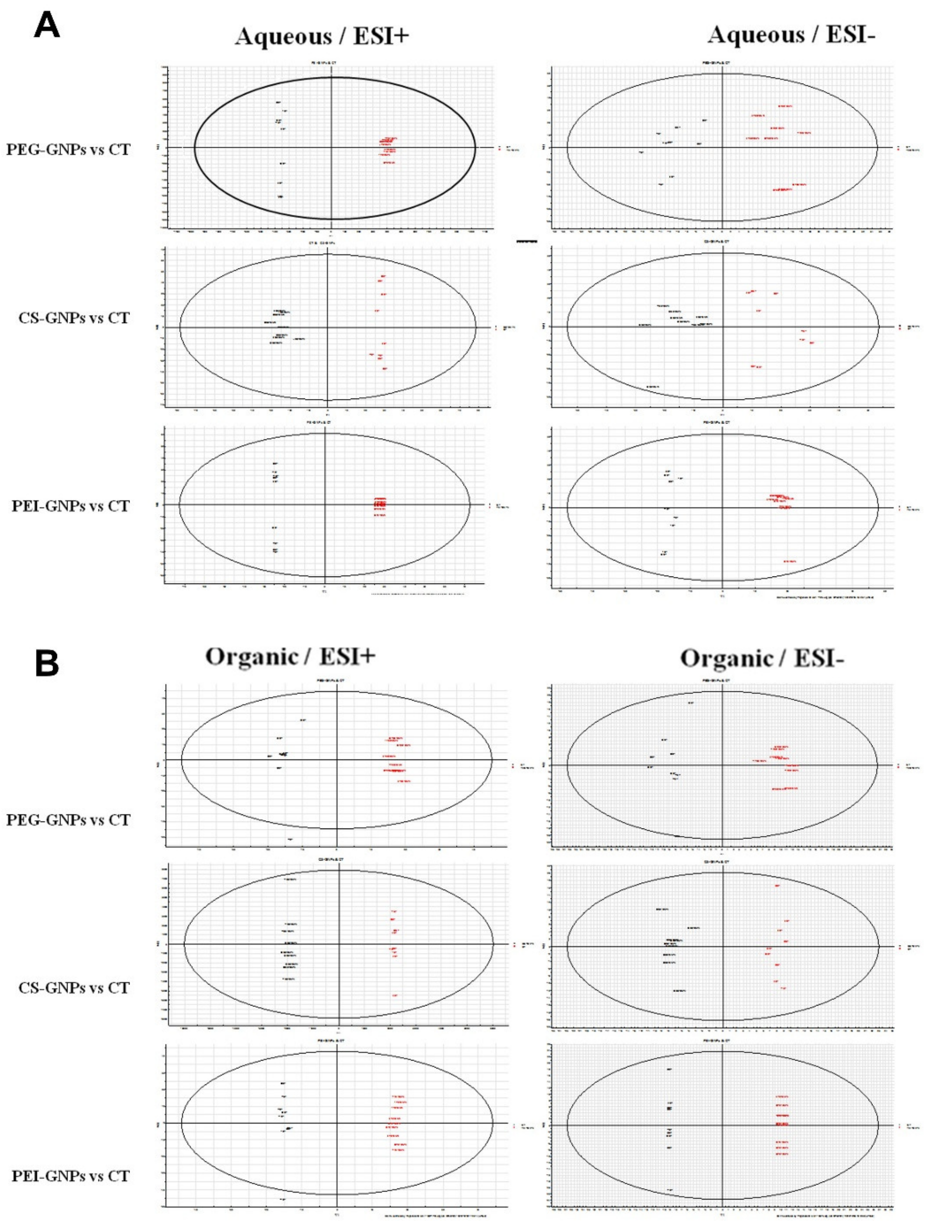

Figure S3. Two-dimensional OPLS-DA score plot of metabolic patterns in the PEG-GNPs, CS-GNPs, and PEI-GNPs injection groups. (A) Aqueous phase extract. (B) Organic phase extract.

Table S1. Differently expressed gene and metabolite.

Pathway Name	P	FDR	Impact	Compound ID/Name
**PEG-GNPS**				
Sphingolipid metabolism	0.0000	0.0000	0.3083	C00550/ Sphingomyelin; C00195 / N-Acylsphingosine; C01190 / Glucosylceramide
Glycerophospholipid metabolism	0.0000	0.0000	0.1990	C00157 / Phosphatidylcholine; C00350 / Phosphatidylethanolamine
Porphyrin and chlorophyll metabolism	0.0081	0.0091	0.0902	C02191 / Protoporphyrin
Glycosylphosphatidylinositol (GPI)-anchor biosynthesis	0.0057	0.0085	0.0040	C00350 / Phosphatidylethanolamine
**CS-GNPs**				
Ether lipid metabolism	0.0001	0.0006	0.1446	C03820/1-Alkyl-2-acetyl-sn-glycerol; C04317 / 1-Organyl-2-lyso-sn-glycero-3-phosphocholine
Porphyrin and chlorophyll metabolism	0.0001	0.0006	0.0902	C02191 / Protoporphyrin
Glycerophospholipid metabolism	0.0002	0.0006	0.2163	C00157 / Phosphatidylcholine; C00350 / Phosphatidylethanolamine; C04230 / 1-Acyl-sn-glycero-3-phosphocholine
Sphingolipid metabolism	0.0002	0.0006	0.2170	C06126 / Digalactosylceramide; C02686 / Galactosylceramide; C01190 / Glucosylceramide
Glycosylphosphatidylinositol (GPI)-anchor biosynthesis	0.0002	0.0006	0.0040	C00350 / Phosphatidylethanolamine
Retinol metabolism	0.0007	0.0011	0.2455	C00376 / Retinal
Pantothenate and CoA biosynthesis	0.0037	0.0042	0.2357	C01134 / Pantetheine 4'-phosphate
Steroid hormone biosynthesis	0.0045	0.0052	0.0132	C05490 / 11-Dehydrocorticosterone; C05488 / 11-Deoxycortisol
Glutathione metabolism	0.0114	0.0114	0.2560	C00051 / Glutathione
**PEI-GNPS**				
Ether lipid metabolism	0.0000	0.0000	0.1446	C03820 / 1-Alkyl-2-acetyl-sn-glycerol ; C04317 / 1-Organyl-2-lyso-sn-glycero-3-phosphocholine
PorSphingolipid metabolism	0.0000	0.0000	0.5051	C00836 / Sphinganine; C00195 / N-Acylsphingosine; C06126 / Digalactosylceramide; C01190 / Glucosylceramide; C12144 / Phytosphingosinephyrin and chlorophyll metabolism
Glycerophospholipid metabolism	0.0006	0.0017	0.2163	C00157 / Phosphatidylcholine; C00350 / Phosphatidylethanolamine; C04230 / 1-Acyl-sn-glycero-3-phosphocholine
Glycosylphosphatidylinositol (GPI)-anchor biosynthesis	0.0017	0.0038	0.0040	C00350 / Phosphatidylethanolamine
Porphyrin and chlorophyll metabolism	0.0038	0.0070	0.0902	C02191 / Protoporphyrin
Pyruvate metabolism	0.0055	0.0087	0.0591	C03451 / (R)-S-Lactoylglutathione
Glutathione metabolism	0.0096	0.0132	0.2560	C00051 / Glutathione
